# Chemistry and Biology of Siderophores from Marine Microbes

**DOI:** 10.3390/md17100562

**Published:** 2019-09-29

**Authors:** Jianwei Chen, Yuqi Guo, Yaojia Lu, Bixia Wang, Jiadong Sun, Huawei Zhang, Hong Wang

**Affiliations:** 1 College of Pharmaceutical Science & Collaborative Innovation Center of Yangtze River Delta Region Green Pharmaceuticals, Zhejiang University of Technology, Hangzhou 310014, China; cjw983617@zjut.edu.cn (J.C.); m17816038135_1@163.com (Y.G.); lyj512637@zjut.edu.cn (Y.L.); 15757116051@163.com (B.W.); hwzhang@zjut.edu.cn (H.Z.); 2 Laboratory of Bioorganic Chemistry, National Institute of Diabetes and Digestive and Kidney Diseases (NIDDK), National Institutes of Health, Bethesda, MD 20878, USA; jiadong.sun@nih.gov

**Keywords:** siderophores, marine microbes, synthesis, biosynthesis

## Abstract

Microbial siderophores are multidentate Fe(III) chelators used by microbes during siderophore-mediated assimilation. They possess high affinity and selectivity for Fe(III). Among them, marine siderophore-mediated microbial iron uptake allows marine microbes to proliferate and survive in the iron-deficient marine environments. Due to their unique iron(III)-chelating properties, delivery system, structural diversity, and therapeutic potential, marine microbial siderophores have great potential for further development of various drug conjugates for antibiotic-resistant bacteria therapy or as a target for inhibiting siderophore virulence factors to develop novel broad-spectrum antibiotics. This review covers siderophores derived from marine microbes.

## 1. Introduction

Marine microbial community diversity and chemodiversity lead to the generation of numerous biological secondary metabolites. These compounds increase the chances of finding valuable drug candidates [1,2,3]. Marine siderophores are a type of low molecular weight natural products, the functions of which are to facilitate the microbial acquisition of iron in the ocean environment [4,5,6]. Marine microbes typically require micromolar levels of iron for their growth, yet the concentration of iron in the surface water of the world ocean is only 0.01–2 nM [7]. In order to overcome this lack of iron, marine microbes have evolved siderophore-mediated delivery system to obtain iron. Siderophores coordinated to insoluble Fe(III) are first transported into microbial cells by membrane bound iron-siderophore receptors. Afterwards, iron is then released from siderophores, typical via reduction of Fe(III) to Fe(II) by microbe-mediated redox processes [8,9,10]. Based on the efficient siderophore-mediated iron acquisition mechanisms, marine siderophores may be developed as novel antimicrobials by covalently attaching clinical antibiotics to marine siderophores or novel iron chelator desferals [11,12,13,14]. Furthermore, marine siderophores have also exhibited various non-classical biological functions, for example, as agents which may interfere with quorum sensing regulation and swarming in bacteria, as mediators of mutualistic interactions, and as secreted signaling molecules regulating virulence factors of pathogens [15,16,17]. These wide range of activities exhibited their great potential in medicine. The present review gives a comprehensive overview of siderophores from marine microorganisms (Table 1).

## 2. Diversity of Siderophores from Marine Microorganisms

**Figure 1:**
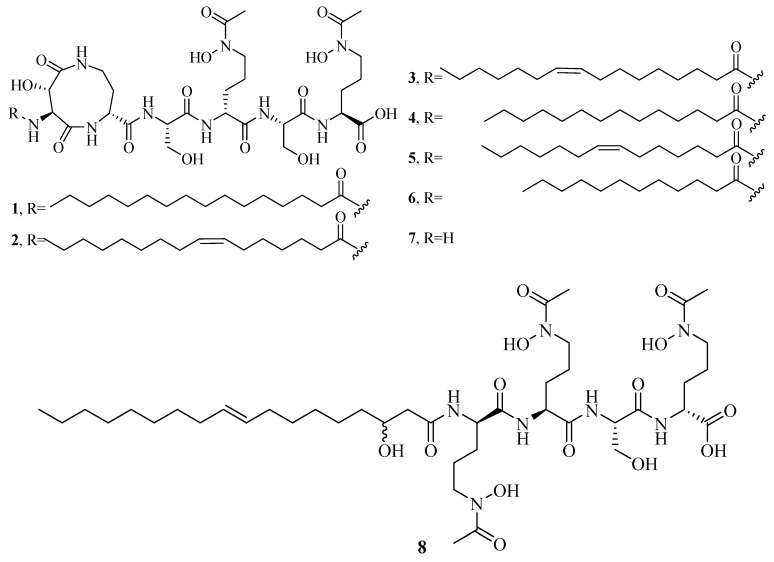

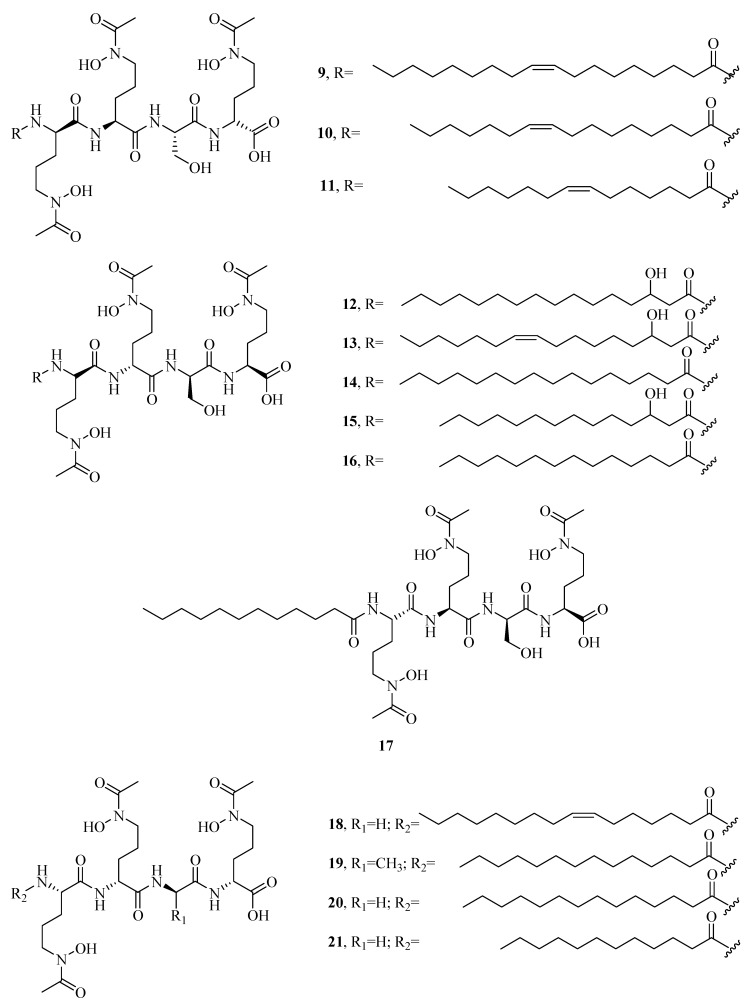

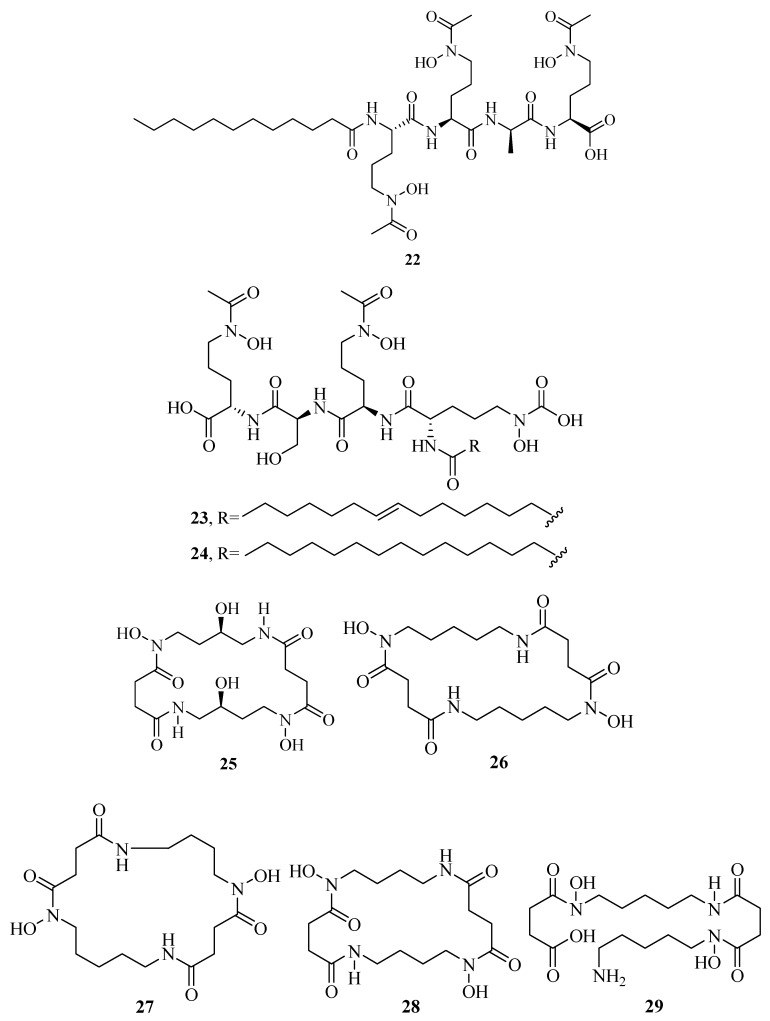

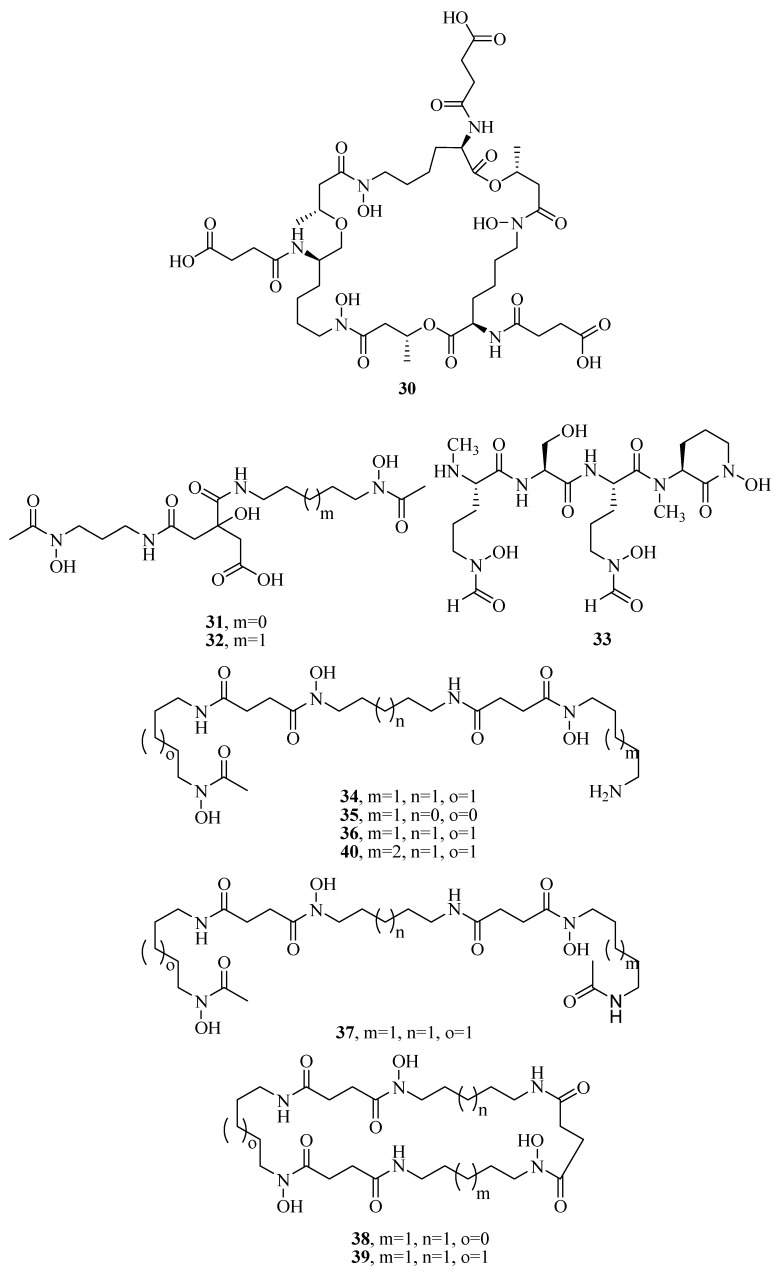
Hydroxamate-type siderophores from marine microbes.

**Figure 2:**
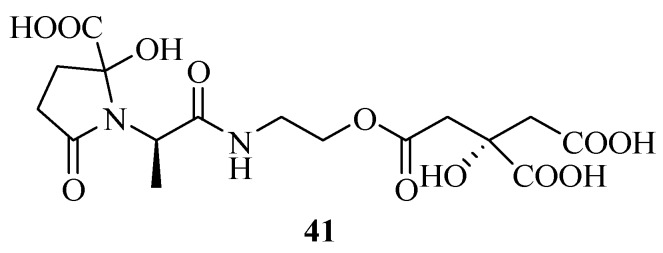
α-Hydroxycarboxylate-type siderophores from marine microbes.

**Figure 3:**
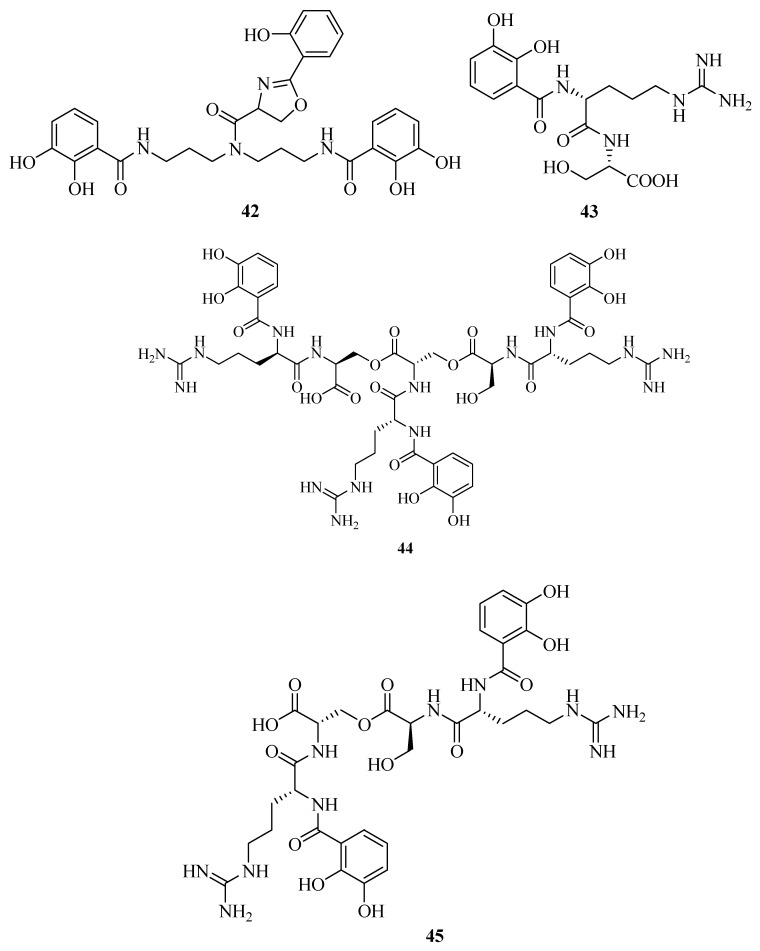

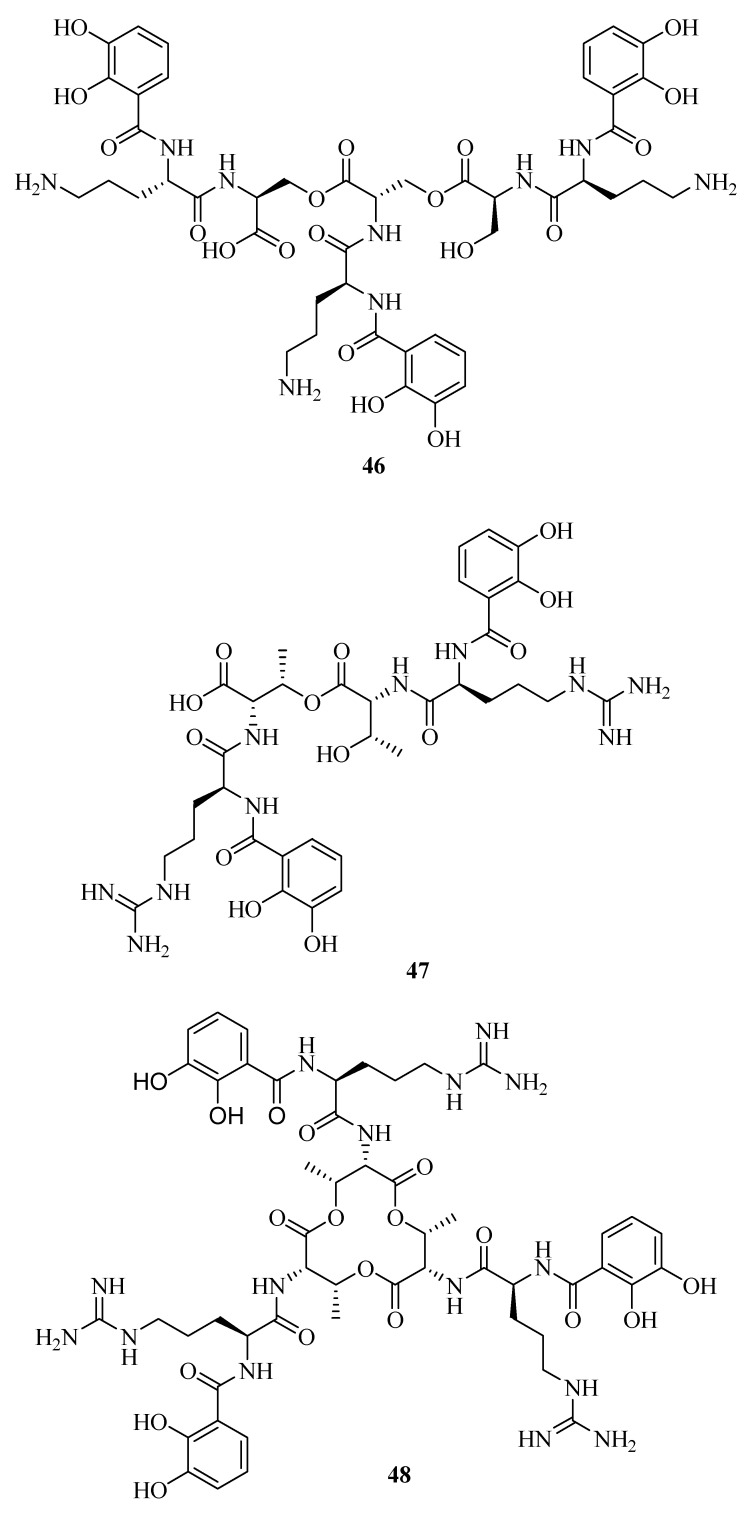

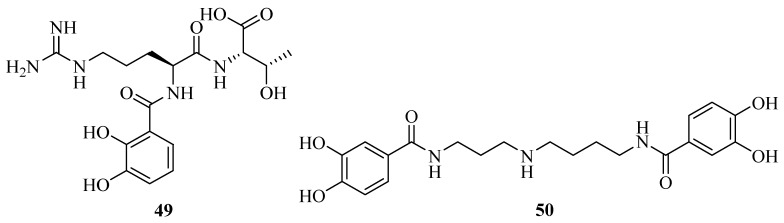
Catecholate-type siderophores from marine microbes.

**Figure 4:**
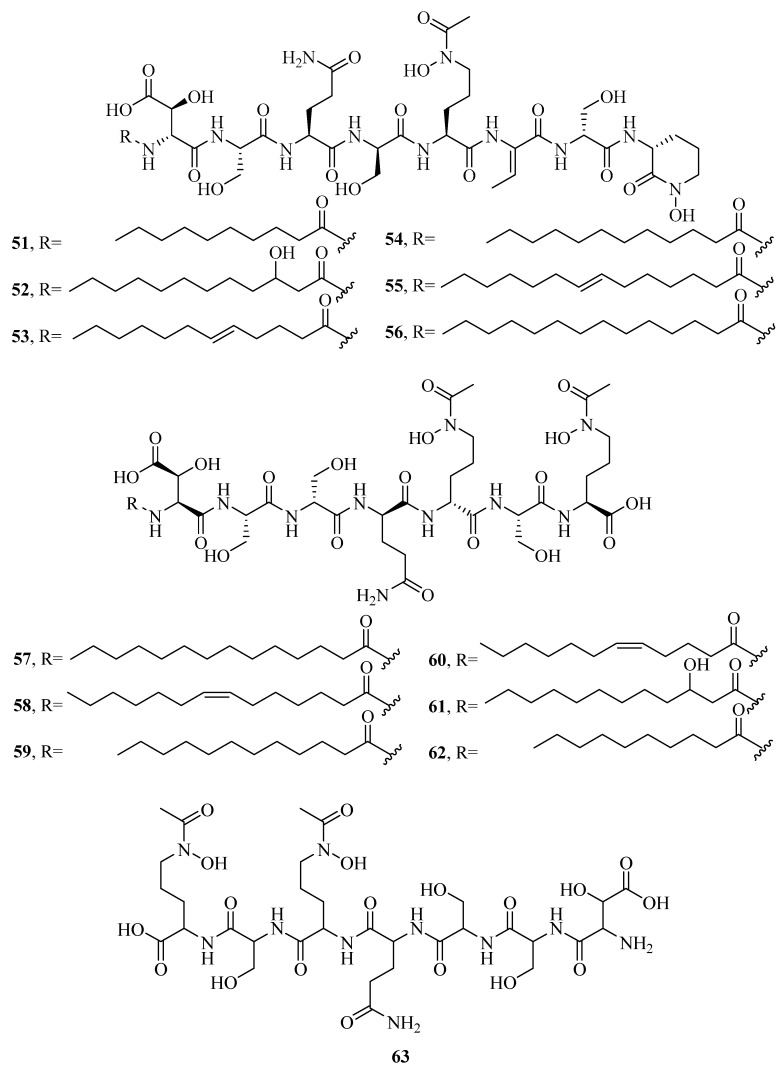

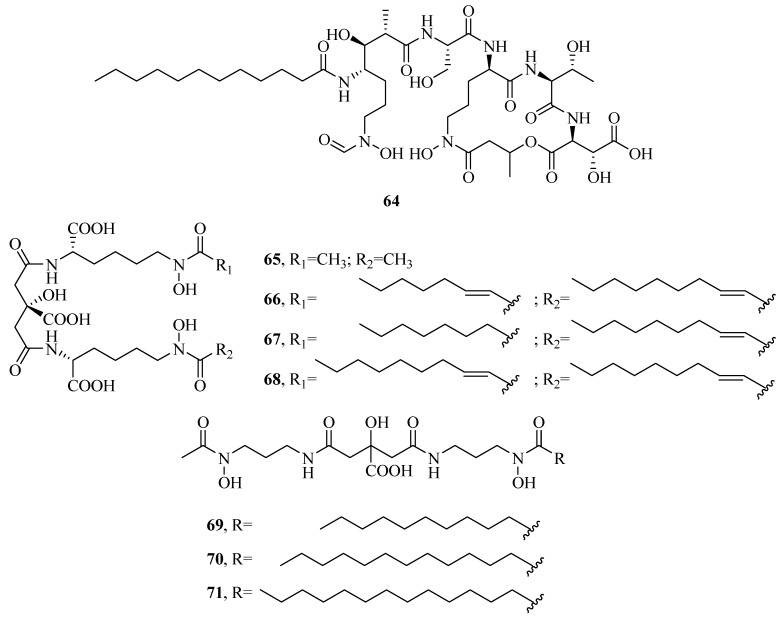
Mixed hydroxamates/α-hydroxycarboxylates siderophores from marine microbes.

**Figure 5:**
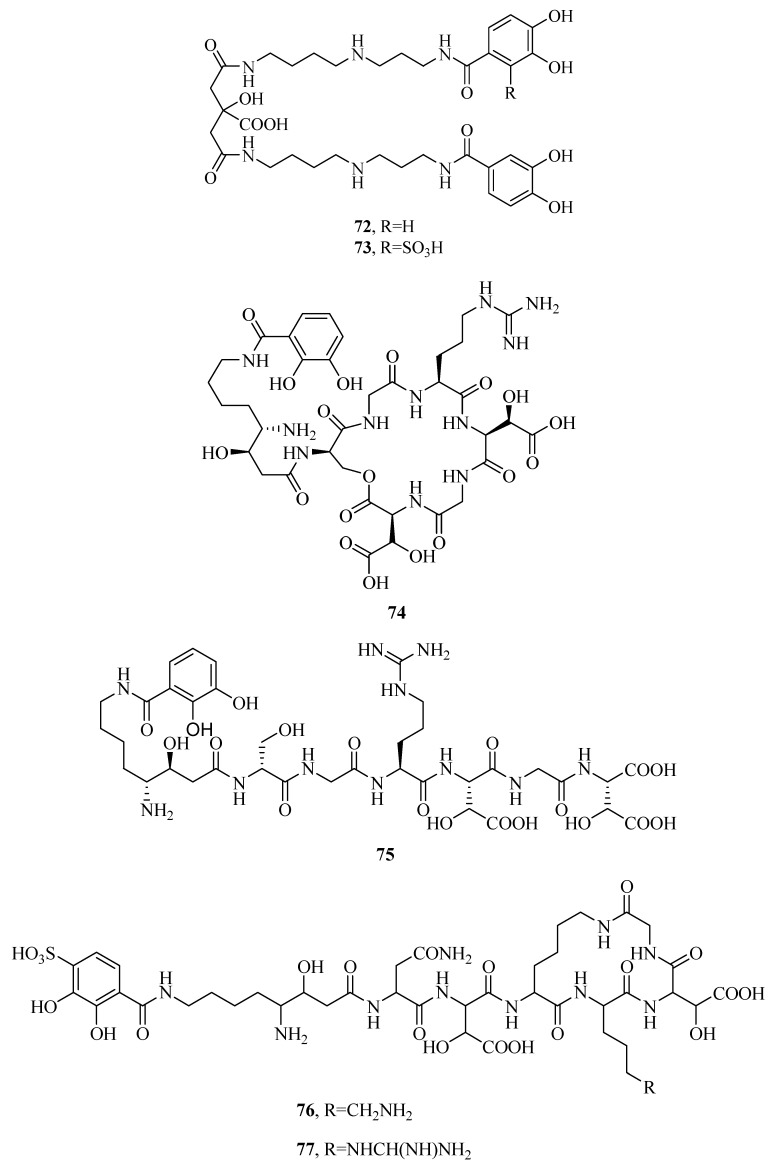
Mixed α-hydroxycarboxylates/catecholates siderophores from marine microbes.

**Figure 6:**
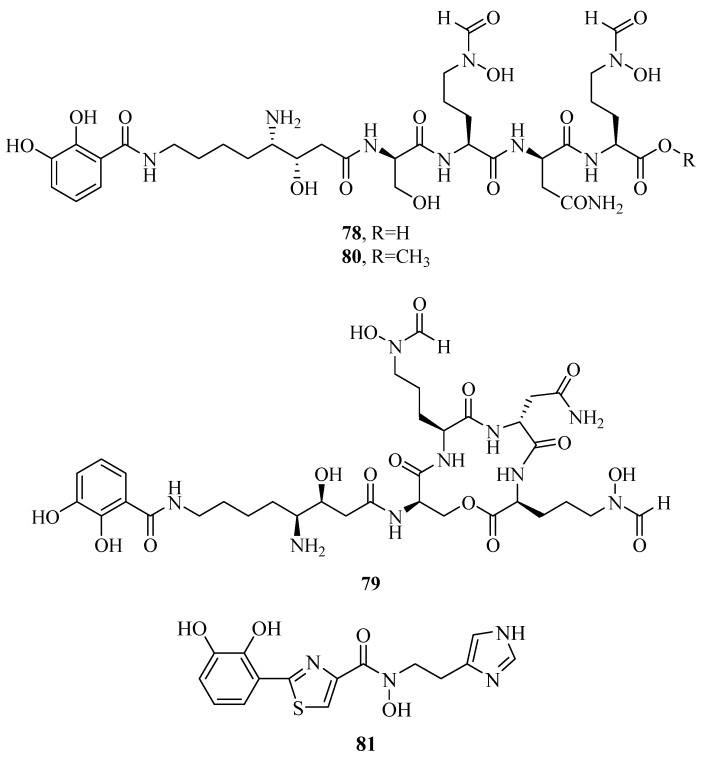
Mixed hydroxamates/catecholates siderophores from marine microbes.

**Figure 7:**
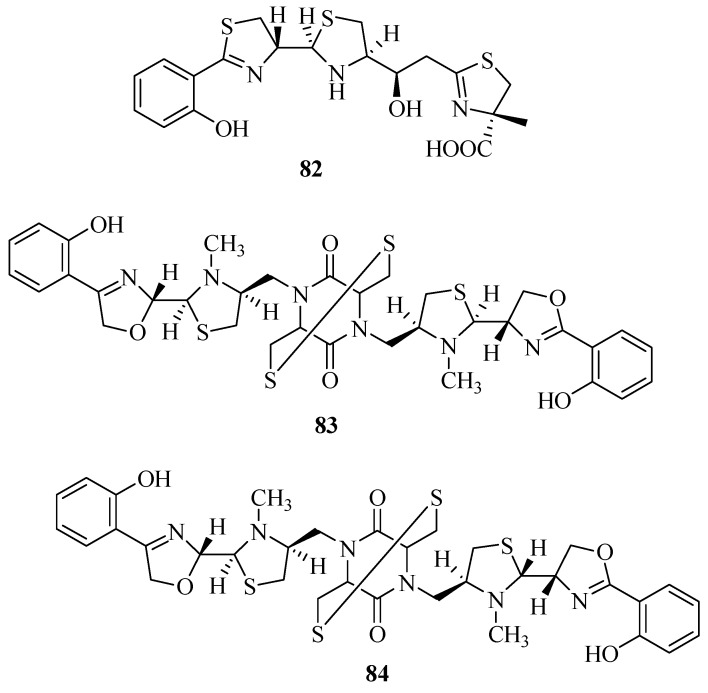
Other types of siderophores from marine microbes.

Recently, a large number of siderophores with diverse structures have been discovered in the marine microorganisms (Table 2). According to their functional groups and hydrophobicity, siderophores from marine microorganisms are divided into seven types, including hydroxamates (Figure 1), α-hydroxycarboxylates (Figure 2), catecholates (Figure 3), mixed hydroxamates/α-hydroxycarboxylates (Figure 4), mixed α-hydroxycarboxylates/catecholates (Figure 5), mixed hydroxamates/catecholates (Figure 6), and other types of siderophores (Figure 7) [18,19,20,21].

### 2.1. Hydroxamate-Type Siderophores

The distinct characteristic of some acyl peptidic hydroxamate-type siderophores is the presence of a nonpolar fatty acid tail. Many marine bacteria have been reported to produce large suites of acyl peptidic hydroxamate siderophores. Marine bacterium *Marinobacter* sp. DS40M6 produces the suit of marinobactin siderophores A–F (**1**–**6**) and HG (**7**) [22,23]. The conversion of exogenously added ^15^N-labeled **1** to the bacterial culture suggests that bacteria produce **7** via the hydrolysis of the acyl amide bond of **1** rather than as a precursor to the compounds **1**–**6** or independent production. It is worth noting that compound **7** can still coordinate Fe(III) via three iron-coordinating moieties, as these moieties are not affected by deacylation. Marine bacterium *Vibrio* sp. R-10 is also reported to produce a suite of amphiphilic siderophores, amphibactins (**8**–**17**) [24]. Each amphibactin has the similar structure, including a peptidic headgroup composed of one serine residue and three ornithine residues but differs in the saturated, unsaturated, or hydroxylated acyl appendage ranging from C-14 to C-18. They are cell-associated siderophores. This association may play an important role in cell defense against siderophore diffusion in the marine environments. Moreover, several acyl peptidic hydroxamate siderophores, moanachelins (**18**–**22**) [25] from the marine bacterium, *Vibrio* sp. Nt1, and amphibactins U–V (**23**–**24**) [26] from two marine bacterial species (*Synechococcus* sp. PCC 7002 and *Vibrio cyclitrophicus* 1F53) have also been identified.

Additionally, several macrocyclic hydroxamate siderophores were discovered in the marine bacteria, such as alcaligin (**25**) [27], bisucaberin (**26**) [28], avaroferrin (**27**) [29], and putrebactin (**28**) [30]. Alcaligin (**25**) could promote Fe(III) uptake in the iron limited cells. It was originally discovered from *Alcaligenes denitrificans* isolated from sediments of lake Biwa, Japan [31]. Recently, it was also obtained from *Alcaligenes eutrophus* CH34 [27]. Bisucaberin (**26**) was isolated from marine bacterium *Alteromonas haloplanktis* SB-1123 [28]. It could inhibit the growth of both invasive micropapillary carcinoma and L-1210 leukemia cells with IC_50_ of 12.7 and 9.7 μM, respectively, and sensitize tumor cells to macrophage-mediated cytolysis [32]. Studies of ecology showed that the increasing contents of bisucaberin (**26**) and acidification enhanced the solubilities and dissolution rates of ferric hydroxides. This suggested that siderophores generated by marine bacteria could grasp ferric hydroxides in aeolian particles to meet the growth of phytoplankton in the ocean environment. Thus, the production rate of marine siderophores may play a vital role in the survival of phytoplankton and biogeochemical cycle of iron in the sea environment [33]. As an open form of **26**, a linear dimeric hydroxamate class siderophore, bisucaberin B (**29**) was isolated from the marine bacterium *Tenacibaculum mesophilum* collected in the Republic of Palau [34]. Its ferric ion-chelating activity was consistent with that of its macrocyclic counterpart **26**. Moreover, the siderophore avaroferrin (**27**) was recently identified in the marine bacterium *Shewanella algae* B516 by heterologous expression of deep-sea sediment metagenomics DNA [29]. It is a macrocyclic heterodimer of *N*-hydroxy-*N*-succinyl-putrescine and *N*-hydroxy-*N*-succinyl cadaverine that is shown to inhibit the swarming of *Vibrio alginolyticus* B522. Another new cyclic trihydroxamate compound, thalassosamide (**30**), was isolated from the marine-derived bacterium *Thalassospira profundimaris* [35] and showed moderate antibiotic activity against *Escherichia coli* and *Pseudomonas aeruginosa* with the same MIC value of 64 μg/mL in vitro. In vivo antibacterial assays showed thalassosamide (**30**) could reduce the infection of *P. aeruginosa* without toxicity compared with untreated control animals in the murine thigh *P. aeruginosa* infection model.

New bioactive siderophores, fradiamine A (**31**) and fradiamine B (**32**), were isolated from the *Streptomyces fradiae* MM456M-mF7 derived from the marine sediments of Calyptogena Community, Sagami Bay, Japan. They contain two alkyl amines asymmetrically bonded to citrate, rarely observed in siderophores. These molecules exhibited medium antibiotic activity against *Clostridium difficile* BAA-1382 of IC_50_ values of 32 and 8 μg/mL, respectively. However, their antimicrobial activities were cancelled dose-dependently under the presence of Fe(III) [36]. A novel albisporachelin siderophore (**33**) was obtained from iron-depleted culture broths of marine actinomycete *Amycolatopsis albispora* WP1^T^. The study of iron-chelating ability suggested that its hydroxamate moieties were involved in iron binding, which was consistent with its structure [37]. Moreover, the variable siderophore metabolome of the marine actinomycete *Salinispora tropica* CNB-440 revealed the production of six known siderophores, desferrioxamine A_1_, A_2_, B, D_1_, D_2_, and E (**34**–**39**), and one novel siderophore desferrioxamine N (**40**) [38]. Among these, **36** has been developed as an iron chelator desferal for treating iron overload in man.

### 2.2. α-Hydroxycarboxylates

Vibrioferrin (**41**) was originally isolated from an enteropathogenic estuarine bacterium, *Vibrio parahaemolyticus*. Recently, it was also obtained from *Marinobacter* species collected from Pacific and Atlantic Oceans. **41** was considered as one of the weakest iron chelators of known marine siderophores according to the metal–ligand binding constant for **41**–Fe(III) (10^24.02(5)^). However, **41**–Fe(III) complex was shown to be more sensitive to photolysis than other marine photoactive siderophores, such as aerobactin, petrobactin, marinobactins, or aquachelins. Taken together, **41** photodegradation may be an evolutionarily adaptive response by which the marine bacteria share photochemically produced soluble iron with their algal associates possibly through the exchange with algal-excreted metabolites [39].

### 2.3. Catecholates

Structural elucidation revealed the discovery of a novel siderophore, nigribactin (**42**) derived from marine bacteria *Vibrio nigripulchritudo* [40]. It could enhance the expression of *spa* encoding a major surface bound virulence factor, Protein A by inducing *spa* transcription. However, another potent siderophore, enterobactin failed to affect the expression of *Staphylococcus aureus* virulence genes. This suggested the influence of **42** on *spa* expression might be independent from its siderophore activity. Vanchrobactin (**43**) from the marine bacteria *Vibrio anguillarum* is a dipeptide composed by arginine and serine residues linked to the 2,3-dihydroxybenzoyl moiety [41,42,43]. Its catecholate and salicylate groups are two potential bidentate coordination sites of Fe^3+^ [44]. The marine bacterium *Vibrio* sp. DS40M4 was reported to produce **43**, a new triscatechol amide siderophore, trivanchrobactin (**44**), and a new related siderophore, divanchrobactin (**45**) [45]. Among them, **43** and **45** may be derived from the hydrolysis products of **44**. All compounds were found to be non-cytotoxic against P388 murine leukemia cell lines. In addition, a novel triscatecholate siderophore, turnerbactin (**46**) was isolated from the shipworm endosymbiont *Teredinibacter turnerae* T7901 [46]. It is a trimer of *N*-(2,3-dihydroxybenzoic acid)-l-Orn-l-Ser with three monomeric units through the Ser ester linkages. Its structure was similar to the catecholate siderophore **43**. They have a serine backbone, 2,3-dihydroxybenzoic acid functional moiety, a positively charged, and a hydrophilic spacer amino acid.

Using the chrome azurol sulfonate (CAS) assay-guided isolation, three new siderophores, dibenarthin (**47**), streptobactin (**48**), and tribenarthin (**49**) were isolated from the marine actinomycete *Streptomyces* sp. YM5-799 collected from Hokkaido in north Japan. Among these, the ED_50_ values of **47** and **48** were 117 μM and 156 μM, slightly stronger than that of the control, deferoxamine mesylate (ED_50_ = 195 μM), whereas **49** exhibited a weak binding with Fe(III) (ED_50_ = 937 μM). These siderophores might play a key role in the survival of marine-derived *Streptomyces* sp. YM5-799 under iron-limited conditions [47]. The marine *Penicillium bilaii* from Port Huon, Tasmania, Australia was also reported to produce a rare catechol-type siderophore, pistillarin (**50**) [48]. It showed potent iron chelation as well as antioxidant activity (free radical-scavenging activity), and significantly protected DNA against the damage of hydroxyl radicals generated by the Fenton reaction [49].

### 2.4. Mixed Hydroxamates/α-Hydroxycarboxylates

Many of the marine siderophores are amphiphilic, including a polar head group and a nonpolar fatty acid tail, which allow them to form micelles or to be tethered to the bacterial outer cell membranes to avoid the problem of fast diffusion of free siderophores without binding to iron [50]. A suite of amphiphilic siderophores, loihichelins A–F (**51**–**56**), are isolated from marine bacterium *Halomonas* sp. LOB-5 [51]. They are comprised of a hydrophilic headgroup (an octapeptide consisting of cyclic *N*(*δ*)-hydroxy-d-ornithine, d-serine, dehydroamino-2-butyric acid, l-*N*(*δ*)-acetyl-*N*(*δ*)-hydroxyornithine, l-serine, l-glutamine, d-serine, and d-*threo*-*β*-hydroxyaspartic acid), and different length of fatty acid tail, ranging from decanoic acid to tetradecanoic acid. A curious characteristic of the loihichelins is their photoreactivity. However, Halomonas LOB-5 is obtained from sediments below a depth of 1714 m, where sunlight would not penetrate. Thus, the photoreactivity of the Fe(III)-loihichelins may not be mainly responsible for Fe(III) uptake. Halomonas aquamarina DS40M3, isolated from the sample of seawater under iron limited and pure culture conditions led to the biosynthesis of aquachelin siderophores A–D (57–60), I (61), and J (62) [22,23,52]. Among them, 61 and 62 were more hydrophilic than 57–60. When H. aquamarina DS40M3 and Marinobacter sp. DS40M6 were co-cultured, a novel aquachelin siderophore HG (63) from hydrolysis of acyl aquachelins was produced. However, acyl amidase activity of *Marinobacter* sp. DS40M6 is predominantly constrained to the cellular membrane. Thus, the acyl aquachelins within the *Marinobacter* membrane are mainly responsible for aquachelin HG (**63**) production [29]. An amphiphilic siderophore imaqobactin (**64**) is detected in the arctic marine bacterium *Variovorax* sp. RKJM285 [53]. Its structure has been implicated in the photoreduction of Fe(III) to Fe(II). Reduction may occur through a charge transfer from ligand-to-metal leading to the cleavage of **64**. Metal-binding capabilities showed that **64** was capable of forming stable adducts with Fe(III), Ga(III), and Al(III). Moreover, **64** also appeared to be reactive with gold, but more studies were required to determine whether this was a reductive process. Of particular note, Fe(III)–**64** and Ga(III)–**64** salts showed no antimicrobial activity. However, **64** showed moderate antimicrobial activity against *Staphylococcus warneri*, methicillin-resistant *S. aureus*, vancomycin-resistant *Enterococcus* and *Proteus vulgaris* with the IC_50_ values of 28, 35, 11, and 14 μM, respectively. It may be attributed to the ability of **64** to bind Fe(III) or Ga(III), depressing microbial proliferation via cellular iron depletion.

The planktonic marine *Vibrio* sp. DS40M5 was reported to produce a mixed hydroxamates/α-hydroxycarboxylate siderophore, aerobactin (**65**) [54]. Subsequently, other marine environmental strains of *Vibrio* also were reported to produce **65**. It was a shuttle molecule recycled in each cycle of transport [55]. Recently, three **65**-based amphiphilic siderophores, ochrobactins A–C (**66**–**68**) were discovered from the marine proteobacterium *Ochrobactrum* sp. SP18. They contain a citric backbone, two lysine residues and different fatty acids. This is the first example of aerobactin-derivative siderophores with two different fatty acid appendages. The ochrobactin–Fe(III) complexes are photoreactive under visible sunlight or UV light. Fe(II) is produced by the photoreaction of ochrobactin–Fe(III) [56]. Moreover, three siderophores, synechobactins (**69**–**71**) from the marine cyanobacteria *Synechococcus* sp. PCC 7002 were identified by a high throughput method which is combined liquid chromatography inductively coupled plasma mass spectrometry with high resolution electrospray ionization mass spectrometry [57].

### 2.5. Mixed α-Hydroxycarboxylates/Catecholates

Two photoreactive siderophores, petrobactin (**72**) and petrobactin sulfonate (**73**), are produced by marine bacterium *Marinobacter hydrocarbonoclasticus* [58,59,60]. They are composed of a citrate bis-spermidine skeleton, with two 3,4-dihydroxy-dihydroxybenzoyl groups providing four donor groups for Fe(III) binding. Fe(III) complexes with **72** and **73** are photoreactive under visible sunlight, regulated by the Fe(III)–citrate moiety. This reaction leads to decarboxylation and reduction of Fe(III) to Fe(II). **73** is also the first marine siderophore including a sulfonated 3,4-dihydroxy aromatic ring. Alterobactin A (**74**) and B (**75**) was originally obtained from *Alteromonas luteoviolacea* isolated by oligotrophic and coastal waters. Alterobactin A (**74**) showed an exceptionally high affinity for Fe(III) [61,62]. It contains two unusual amino acids: (3*R*,4*S*)-4,8-diamino-3-hydroxyoctanoic acid and l-threo-β-hydroxyaspartic acid, attaching to a catechol carboxylate. **74**–Fe(III) complex is stable in solution with little change of absorbance. However, in the absence of coordinated Fe(III), the serine ester of **74** will be hydrolyzed to alterobactin B (**75**) siderophore. New siderophores, pseudoalterobactin A (**76**) and B (**77**) were obtained from marine bacterium *Pseudoalteromonas* sp. KP20-4 isolated from the Republic of Palau [63]. They contain a catechol and two β-hydroxy-Asp residues. Pseudoalterobactins showed strong activity by a CAS assay, which was comparable to that of desferrioxamine B and enterobactin. Both **76** and **77** exhibited ED_50_ value of 20 μM (the reduction of the absorbance at 630 nm of the CAS solution by 50%), while desferrioxamine B and enterobactin showed ED_50_ values of 500 μM and 60 μM under the same conditions, respectively.

### 2.6. Mixed Hydroxamates/Catecholates

Three unusual siderophores, lystabactins A–C (**78**–**80**), were obtained from the marine bacterium *Pseudoalteromonas* sp. S2B [64]. In order to further determine their stability by chelating Fe(III), the pFe scale was used to compare with the iron-binding stability of siderophores. pFe was the negative logarithm of aqueous, free Fe(III) at fixed concentrations of siderophore, Fe(III), and acidification (pH). The tighter a given siderophore bound Fe(III), the higher the pFe. The pFe of compounds **78** and **79** were calculated to be 26.0 and 27.5, respectively. This suggested that their stability of iron-binding was stronger than the tris-hydroxamate siderophore, desferrioxamine B, while weaker than the tris-catecholate siderophore, enterobactin. Anguibactin (**81**) siderophore was found in the marine pathogen *V. anguillarum* or *Vibrio* sp. DS40M4 [42,45]. It was evaluated cytotoxic effects on the P388 murine leukemia cell lines and was shown to be cytotoxic (IC_50_ < 15 μM). It was also identified as an important virulence factor.

### 2.7. Other Types of Siderophores

Besides, several other types of siderophores were also discovered in the marine microorganisms. A novel siderophore, piscibactin (**82**), was isolated from the marine fish pathogens *Photobacterium damselae* subsp. *piscicida* and *V. anguillarum*. The inactivation of piscibactin system would lead to a severe loss of the virulence degree. Therefore, piscibactin had a great impact in the expression of virulence, the ability to piscibactin seemed to be sufficient to confer maximal virulence to pathogens [65,66,67]. Two new oxazole–thiazole siderophore compounds, tetroazolemycins A (**83**) and B (**84**), were isolated from marine *Streptomyces olivaceus* FXJ8.012 [68]. Their heavy metal ion-binding ability was evaluated by Cu^2+^, Zn^2+^, Fe^3+^, Pb^2+^, Cr^3+^, and Mn^2+^. The results indicated that **82** and **83** had affinity for Cu^2+^, Zn^2+^, and Fe^3+^ but not affinity for Pb^2+^, Cr^3+^, and Mn^2+^. Further studies showed the free-state tetroazolemycins did not show antimicrobial activity. Their metal ion complexes were also inactive against pathogens *E. coli*, *S. aureus*, *Mycobacterium gilvum*, *Bacillus subtilis*, *P. aeruginosa*, *Candida pseudorugosa*, *Candida albicans*, *Rhizoctonia solani*, *Aspergillus fumigatus*, and *Fusarium oxysporum*, inactive against A/H1N1 influenza virus, and inactive against human lung adenocarcinoma cell line A549 and murine macrophage cell line P388D. However, their Zn^2+^ complexes showed weakly activity against pathogenic *Klebsiella pneumoniae* with MICs of 125–250 μg/mL and 125 μg/mL, respectively. Thus, it was speculated that the antimicrobial activity of oxazole/thiazole siderophores might be closely related to Zn^2+^.

**Table 2:** Siderophores from marine microorganisms.

Compd.	Name	PubChem CID	PubChem Database	Ref.
**1**	Marinobactin A	–	–	[22]
**2**	Marinobactin B	–	–	[22]
**3**	Marinobactin C	–	–	[22]
**4**	Marinobactin D	–	–	[22]
**5**	Marinobactin E	–	–	[22]
**6**	Marinobactin F	–	–	[22]
**7**	Marinobactin HG	–	–	[23]
**8**	Amphibactin	–	–	[24]
**9**	Amphibactin	–	–	[24]
**10**	Amphibactin	–	–	[24]
**11**	Amphibactin	–	–	[24]
**12**	Amphibactin	–	–	[24]
**13**	Amphibactin	–	–	[24]
**14**	Amphibactin	–	–	[24]
**15**	Amphibactin	–	–	[24]
**16**	Amphibactin	–	–	[24]
**17**	Amphibactin	–	–	[24]
**18**	Moanachelin	–	–	[25]
**19**	Moanachelin	–	–	[25]
**20**	Moanachelin	–	–	[25]
**21**	Moanachelin	122223347	https://pubchem.ncbi.nlm.nih.gov/compound/122223347	[25]
**22**	Moanachelin	–	–	[25]
**23**	Amphibactin U	–	–	[26]
**24**	Amphibactin V	–	–	[26]
**25**	Alcaligin	–	–	[27]
**26**	Bisucaberin	–	–	[28]
**27**	Avaroferrin	–	–	[29]
**28**	Putrebactin	–	–	[30]
**29**	Bisucaberin B	–	–	[34]
**30**	Thalassosamide	–	–	[35]
**31**	Fradiamine A	129008905	https://pubchem.ncbi.nlm.nih.gov/compound/129008905	[36]
**32**	Fradiamine B	60151746	https://pubchem.ncbi.nlm.nih.gov/compound/60151746	[36]
**33**	Albisporachelin	–	–	[37]
**34**	Desferrioxamine A_1_	–	–	[38]
**35**	Desferrioxamine A_2_	–	–	[38]
**36**	Desferrioxamine B	–	–	[38]
**37**	Desferrioxamine D_1_	–	–	[38]
**38**	Desferrioxamine D_2_	–	–	[38]
**39**	Desferrioxamine E	–	–	[38]
**40**	Desferrioxamine N	–	–	[38]
**41**	Vibrioferrin	11102119	https://pubchem.ncbi.nlm.nih.gov/compound/11102119	[39]
**42**	Nigribactin	–	–	[40]
**43**	Vanchrobactin	–	–	[45]
**44**	Trivanchrobactin	–	–	[45]
**45**	Divanchrobactin	–	–	[45]
**46**	Turnerbactin			[46]
**47**	Dibenarthin	–	–	[47]
**48**	Streptobactin	–	–	[47]
**49**	Tribenarthin	–	–	[47]
**50**	Pistillarin	–	–	[48]
**51**	Loihichelin A	101476230	https://pubchem.ncbi.nlm.nih.gov/compound/101476230	[51]
**52**	Loihichelin B	101476231	https://pubchem.ncbi.nlm.nih.gov/compound/101476231	[51]
**53**	Loihichelin C	101476232	https://pubchem.ncbi.nlm.nih.gov/compound/101476232	[51]
**54**	Loihichelin D	101476233	https://pubchem.ncbi.nlm.nih.gov/compound/101476233	[51]
**55**	Loihichelin E	101476234	https://pubchem.ncbi.nlm.nih.gov/compound/101476234	[51]
**56**	Loihichelin F	101476235	https://pubchem.ncbi.nlm.nih.gov/compound/101476235	[51]
**57**	Aquachelin A	–	–	[22]
**58**	Aquachelin B	–	–	[22]
**59**	Aquachelin C	–	–	[22]
**60**	Aquachelin D	–	–	[22]
**61**	Aquachelin I	–	–	[23]
**62**	Aquachelin J	–	–	[52]
**63**	Aquachelin HG	–	–	[29]
**64**	Imaqobactin	–	–	[53]
**65**	Aerobactin	–	–	[54]
**66**	Ochrobactin A	–	–	[56]
**67**	Ochrobactin B	–	–	[56]
**68**	Ochrobactin C	–	–	[56]
**69**	Synechobactin	122377042	https://pubchem.ncbi.nlm.nih.gov/compound/122377042	[57]
**70**	Synechobactin	122377043	https://pubchem.ncbi.nlm.nih.gov/compound/122377043	[57]
**71**	Synechobactin	122377044	https://pubchem.ncbi.nlm.nih.gov/compound/122377044	[57]
**72**	Petrobactin	–	–	[58]
**73**	Petrobactin sulfonate	–	–	[59]
**74**	Alterobactin A	–	–	[61]
**75**	Alterobactin B	101775921	https://pubchem.ncbi.nlm.nih.gov/compound/101775921	[62]
**76**	Pseudoalterobactin A	11434714	https://pubchem.ncbi.nlm.nih.gov/compound/11434714	[63]
**77**	Pseudoalterobactin B	11788080	https://pubchem.ncbi.nlm.nih.gov/compound/11788080	[63]
**78**	Lystabactin A	–	–	[64]
**79**	Lystabactin B	–	–	[64]
**80**	Lystabactin C	–	–	[64]
**81**	Anguibactin	–	–	[42,45]
**82**	piscibactin	136754132	https://pubchem.ncbi.nlm.nih.gov/compound/136754132	[65,66,67]
**83**	Tetroazolemycin A	–	–	[68]
**84**	Tetroazolemycin B	–	–	[68]

## 3. Biosynthesis of Siderophores from Marine Microorganisms

To date, more than 80 siderophores have been isolated from marine microorganisms, however, only part of siderophore biosynthetic pathways have been reported. A large number of novel siderophore biosynthetic pathways still need to be further studied. Therefore, researchers should focus more on the biosynthesis of siderophores from marine microorganisms to obtain structurally diverse siderophores as drug candidates.

### 3.1. NRPS-Mediated Siderophore Biosynthetic Pathway

**Scheme 1:**
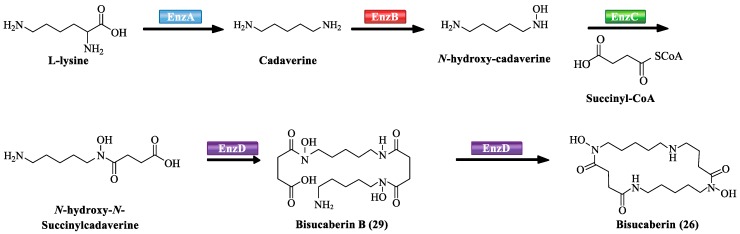
The proposed biosynthesis of bisucaberin B (**29**) and bisucaberin (**26**).

Recently, it has become apparent that the biosynthesis of siderophores involves covalently tethered a series of intermediates to the multienzymes throughout the assembly process and is catalyzed by nonribosomal peptide synthetases (NRPSs). NRPSs are responsible for assembling structurally complex peptides from small building blocks such as carboxyl or amino acids. Each module is highly efficient dedicated to the activation, modification, and incorporation of one small building block into the compound and harbors all key enzymatic activities as specialized domains to catalyze the single chemical reaction (Table 3). The de novo study of biosynthesis showed EnzA–D (NRPSs) were mainly responsible for the biosynthesis of siderophores, bisucaberin B (**29**) and bisucaberin (**26**) (Scheme 1). EnzA and EnzB catalyzed the biosynthesis of intermediates from L-lysine to cadaverine, and cadaverine to *N*-hydroxy-cadaverine, respectively. EnzC activated the acylation of *N*-hydroxy-cadaverine with succinyl-CoA to produce *N*-hydroxy-*N*-succinylcadaverine (HSC) monomer. EnzD as the last step noncovalently bound to HSC and ATP to catalyze the oligomerization–macrocyclization reaction of HSC to form **29** or **26**. Firstly, a HSC molecule and ATP noncovalently bound to the active site of EnzD. A second molecule of HSC also noncovalently bound to EnzD, which dissociated from the active site of EnzD. The activated carboxyl group of the first molecule of HSC underwent a base-promoted addition–elimination reaction with the protonated amino group of the second molecule of HSC to yield the linear dimer **29**. The activated carboxyl group and protonated amino group of **29** could further undergo an intramolecular base-promoted condensation to generate **26** [34,69,70]. In the biosynthetic network of **27**, it exhibits the similar scheme of biosynthesis with **26** and **29**. Firstly, MbsA–C catalyze successive reactions, resulting in ornithine and lysine to the *N*-hydroxy-*N*-succinyl diamines (HSDs). MbsD then utilizes both *N*-hydroxy-*N*-succinyl-putrescine and *N*-hydroxy-*N*-succinyl cadaverine as immediate precursors and catalyzes both the oligomerization and final macrocyclization, leading to the biosynthesis of **27**.

Vibrioferrin **(41)** is synthesized by five biosynthetic enzymes PvsA-E (NRPSs). The biosynthetic pathway starting with enzyme PvsE. PvsE has a highly homologous with 2,6-diaminopimerate decarboxylase, indicating PvsE catalyzes decarboxylation from serine to ethanolamine. PvsB and PvsD form amide bond between L-alanine and 2-ketoglutaric acid, or pentanoic acid and ethanolamine. PvsA is perceived to form an ester bond between ethanolamine and citrate. The final product vibrioferrin **(41)** is secreted by PvsC which is most likely to be a transporter involved in secreting the product from the cell [71].

**Scheme 2:**
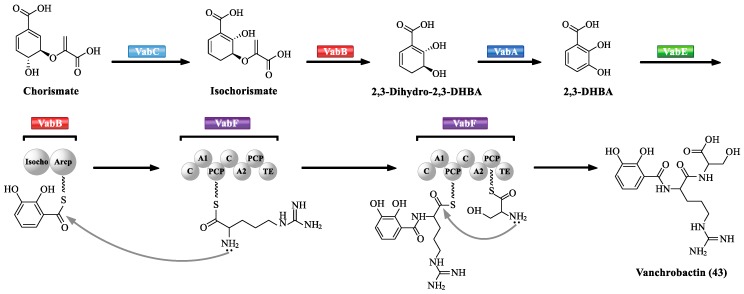
The proposed biosynthesis of vanchrobactin (**43**).

The biosynthetic and regulatory elements of vanchrobactin (**43**) in marine bacteria *V. anguillarum* has been completely elucidated [42,43,44]. It proceeds through a NRPS-mediated pathway encoded by chromosomal genes to produce **43** (Scheme 2). VabB and VabE–F have been identified as three NRPSs. Isochorismate synthase (VabC) first started with the conversion of chorismate into isochorismate. Isochorismatase (VabB) and 2,3-dihydro-2,3-dihydroxybenzoate dehydrogenase (VabA) then catalyzed the biosynthesis of intermediates from isochorismate to 2,3-dihydro-2,3-dihydroxybenzoic acid (2,3-dihydro-2,3-DHBA), and (2,3-dihydro-2,3-DHBA) respectively to 2,3-DHBA. Once 2,3-DHBA was synthesized, VabE (NRPS) activated 2,3-DHBA to its acyl-adenylated intermediate in an ATP-dependent manner, and then VabE (NRPS)transferred this activated intermediate to VabB. VabB further bound with 2,3-DHBA through its ACP domain. VabF (NRPS) was involved in the last steps of **43** assembly. It was composed of two modules, including C-A1-PCP domain (condensation-adenylation-peptidyl-carrier-protein) in the N-terminal, and C-A2-PCP-TE domain (condensation-adenylation-peptidyl-carrier-protein-thioesterase) in the C-terminal. VabF A1 domain activated an arginine residue, and synthesized 2,3-dihydroxybenzoyl-arginine precursor through its PCP domain. VabF A2 domain activated a serine residue. The TE domain as the last domain was to release **43** into solution.

**Scheme 3:**
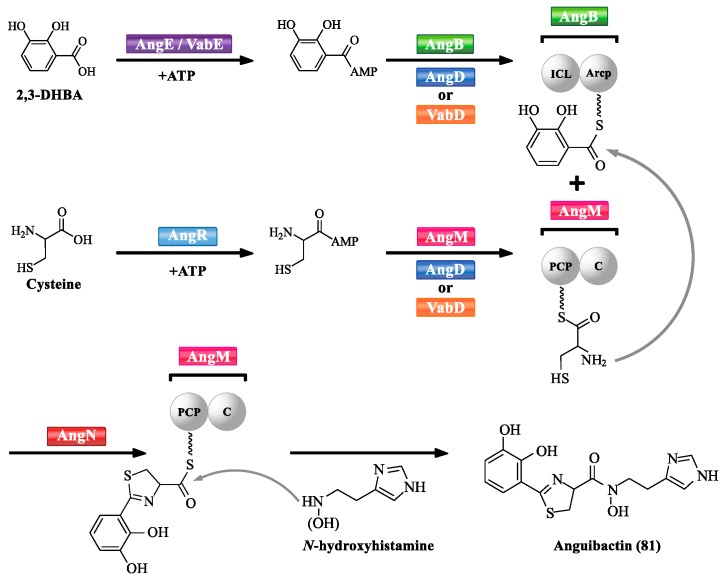
The proposed biosynthetic pathway of anguibactin (**81**).

Mechanism of anguibactin (**81**) biosynthesis has been characterized in *V. anguillarum* (Scheme 3) [72]. A series of NRPSs are involved in *V. anguillarum* anguibactin (**81**) biosynthesis, such as AngB, AngD, AngE, AngM, AngN, and AngR. VabD and VabE are functional homologues of AngD and AngE, respectively. Each protein provides important functional domain(s) for anguibactin (**81**) biosynthesis. The adenylation (A) domains of VabE or likely of AngE activate 2,3-DHBA into 2,3-DHBA-AMP [73,74]. An aryl carrier protein (ArCP) domain of the C-terminal region of AngB is responsible for tethering 2,3-DHBA-AMP. The adenylation (A) domain of AngR is likely related to the activation of cysteine. It is worth noting that cyclisation (Cy) and peptidyl carrier protein (PCP) domains of AngR are not functional due to the replacement of the first aspartic acid by asparagine in the Cy domain, and the replacement of an essential serine by alanine in the PCP domain [75,76]. AngD or VabD is responsible for transferring the phosphopantetheinyl moiety to serine residues located in the ArCP domain of AngB and PCP domain of AngM, making them become active forms [74,77]. The PCP domain of AngM is used to tether the activated cysteine, and its C domain is likely to catalyze peptide bond formation between cysteine from AngM and 2,3-DHBA from AngB. AngN contains two Cy domains, Cy1 and Cy2 that could condense and cyclize 2,3-DHBA and cysteine to produce thiazoline [78,79]. The C domain of AngM could attach *N*-hydroxyhistamine to the dihydroxyphenyl–thiazoline–thioester to form anguibactin (**81**). AngT as the last step is to release anguibactin (**81**) from AngM [72].

**Scheme 4:**
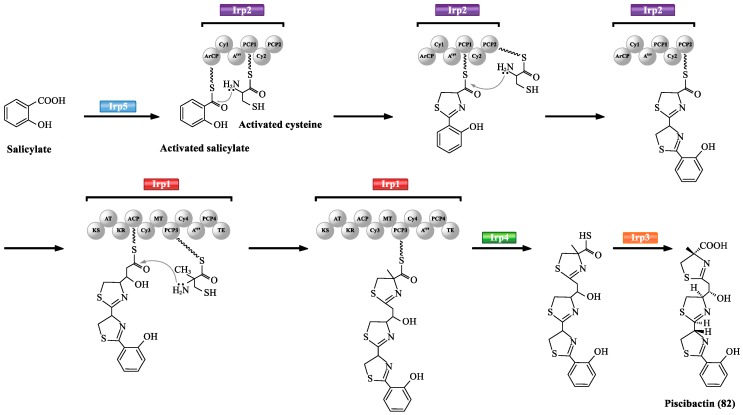
The proposed biosynthesis of piscibactin (**82**).

Irp1-5 of *Photobacterium damselae* subsp. *piscicida* are mainly involved in the biosynthesis of siderophore piscibactin (**82**) (Scheme 4) [65,80]. Firstly, Irp5 (salicylate-activating enzyme) initiates piscibactin assembly, and then transfers the activated salicylate to aryl carrier protein domain of Irp2 (Iron-regulated protein 2). The ArCP domain of Irp2 is specific for cysteine activation. The activated cysteine is transferred to PCP1 and PCP2 simultaneously. Then, Irp2 catalyzes the continuous addition and cyclization of every cycle to form two thiazoline rings connected to a salicylate portion. Irp1 (PKS/NRPS multifunctional enzyme) is composed of 11 domains including a carboxyl-terminated thioesterase domain and three elongation modules. The ketosynthase domain in Irp1 loads a malonyl group which is linked to the ACP domain. The PCP3 domain of Irp1 catalyzes the condensation/heterocyclization of methyl-cysteine. Irp4 (thioesterase) hydrolyzes the thioester linkage. The final maturation of piscibactin (**82**) must be accomplished by Irp3 (reductase) which reduces the middle thiazoline ring to the thiazolidine.

### 3.2. NRPS-Independent Siderophore Biosynthetic Pathway

**Scheme 5:**
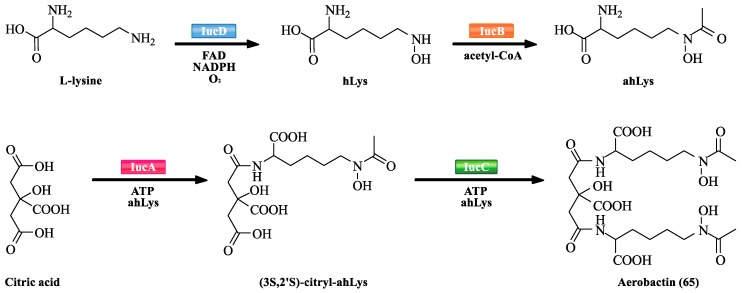
The proposed biosynthetic pathway of aerobactin (**65**).

NRPS-independent siderophore (NIS) synthetases are an emerging member of the family of new synthetases. They do not have the sequence or structural similarity to NRPSs. However, they also play an important role in the biosynthesis of siderophores. Many structurally diverse siderophores are biosynthesized by NIS synthetases (Table 3). NIS synthetases contain type A, type B, and type C based on sequence similarity criteria. Type A enzymes catalyze the condensation of amines or alcohols with a prochiral carboxyl group of citric acid. Type B enzymes catalyze the condensation of amines with γ-carboxyl group of α-ketoglutarate. Type C enzymes catalyze either the oligomerization and macrocyclisation of ω-amino-carboxylic acids, or an amine or alcohol with a monoamide derivative of citric acid. By selective gene knockout and reconstruction of *iuc* genes, aerobactin (**65**) biosynthetic pathway was deciphered. IucA–D are responsible for the biosynthesis of **65** (Scheme 5). They are highly stereoselective. IucB is confirmed to its l-lysine *N^6^* monooxygenase activity [81]. Similarly, IucD is also confirmed to its *N^6^*-hydroxy-l-lysine (hLys) acetyltransferase activity [82]. IucB and IucD respectively catalyze the biosynthesis of intermediates from l-lysine to hLys, and hLys to *N^6^*-acetyl-*N^6^*-hydroxy-l-lysine (ahLys). IucA and IucC represent the archetypal Type A and Type C NIS synthetases, respectively [83]. IucA catalyzes the carboxymethyl group of citric acid to form a citryl-adenylate intermediate via the activation of ATP. IucC then consumes 3*S*,2’*S*-citryl-ahLys intermediate in a relatively modest catalytic efficiency to synthesize **65**.

**Scheme 6:**
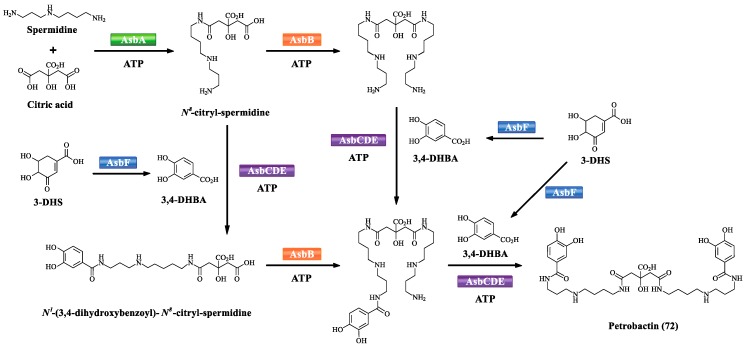
The proposed biosynthetic pathway of petrobactin (**72**).

Based on the NRPS-independent assembly, the biosynthetic scheme of petrobactin (**72**) is developed (Scheme 6). AsbA (type A NIS synthetase) has been demonstrated to catalyze the ATP-dependent condensation of *N^8^* of spermidine with citric acid to afford *N^8^*-citryl-spermidine. Another NIS synthetase AsbB (type C NIS synthetase) catalyzes ATP-dependent condensation of *N^8^*-citryl-spermidine or *N^1^*-(3,4-dihydroxybenzoyl)-*N^8^*-citryl-spermidine with spermidine. AsbF, a 3-dehydroshikimate (3-DHS) dehydratase, catalyzes the conversion of the bacterial metabolite 3-DHS to form 3,4-dihydroxybenzoic acid (3,4-DHBA). AsbC, a NRPS-like adenylating enzyme, is responsible for catalyzing the ATP-dependent acylation of phosphopantetheine thiol of AsbD (a carrier protein) with 3,4-DHBA. AsbE as an acyltransferase is involved in the 3,4-dihydroxybenzoyl group transfer from the carrier protein AsbD to *N^1^* and *N^8^* of spermidine to afford *N^1^*-(3,4-dihydroxybenzoyl)-spermidine and *N^8^*-(3,4-dihydroxybenzoyl)-spermidine, respectively, suggesting that AsbE has relatively relaxed substrate specificity [60,84,85].

## 4. Synthesis and Study of Siderophores from Marine Microorganisms

Marine siderophores, as potentially valuable drug candidates, are attracting extensive attention of researchers. However, the content of siderophores from marine microbes is very low. Therefore, in order to further study their biological activities, mechanisms, absolute configurations, or structure–activity relationships, chemical syntheses of siderophores would be crucial.

### 4.1. Alcaligin

**Scheme 7:**
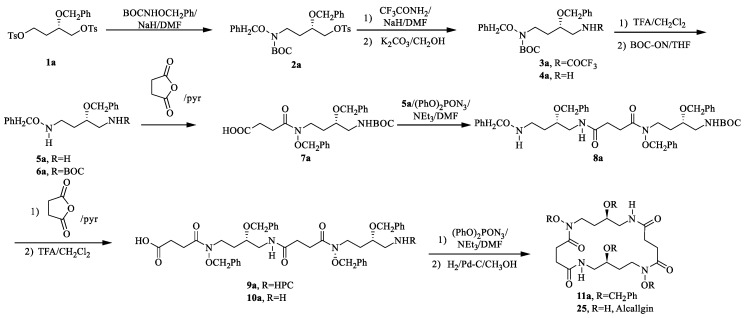
Chemical synthesis of alcaligin (**25**).

Alcaligin (**25**) had similar structural relationship to siderophores bisucaberin (**26**) and nocardamine. Bisucaberin (**26**) could inhibit the growth of both invasive micropapillary carcinoma and L-1210 leukemia cells with IC_50_ of 12.7 and 9.7 μM, respectively, and sensitize tumor cells to macrophage-mediated cytolysis [32]. However, this activity was absent in siderophore nocardamine. An investigation of the biological properties of **25** is particularly attractive. Therefore, Bergeron R.J. et al. [86] first performed the total synthesis of alcaligin (**25**) (Scheme 7). The starting point in the total synthesis of alcaligin was the regiospecific *N*-alkylation of ditosylate **1a** providing monotosylate **2a**. A second amino group was then coupled to C-1 of **2a** by *N*-alkylation of trifluoroacetamide producing the diamide **3a**. The primary amine **4a** was produced by basic cleavage of **3a**. Brief exposure of the *N*-(*tert*-butoxycarbonyl)amine **4a** to trifluoroacetic acid led to the bis(benzyloxy)putrescine **5a**. Addition of 2-[[(*tert*-butoxycarbonyl)oxy]imino]-2-phenylacetonitrile to **5a** resulted in the benzyloxy amine **6a**. **6a** was next acylated with succinic anhydride to generate *tert*-butoxycarbonyl acid **7a**. Subsequently, the condensation of **5a** and **7a** to produce the (benzyloxy)amine **8a**. The second succinate unit was coupled with **8a** to generate *tert*-butoxycarbonyl acid **9a** and **10a**. Macrocyclic tetrabenzyl alcaligin **11a** was obtained under the catalysis of diphenylphosphorazidate. Finally, the benzyl groups of **11a** were removed to obtain alcaligin (**25**).

### 4.2. Vanchrobactin

**Scheme 8:**
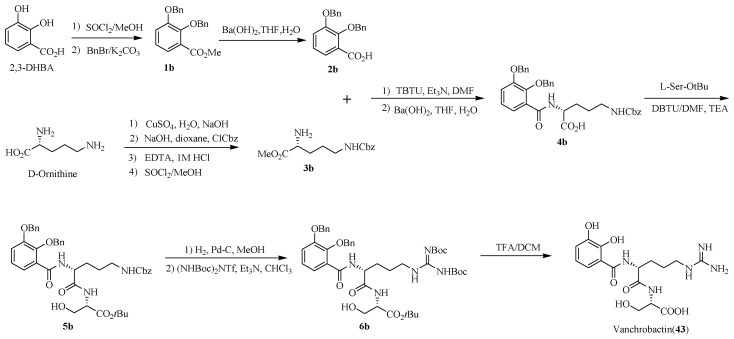
Chemical synthesis of vanchrobactin (**43**).

In the course of the studies into vanchrobactin (**43**), its stereochemistry could not be elucidated by spectroscopic analysis. In order to determine the absolute configuration of vanchrobactin (**43**), Soengas R.G. et al. [87] performed the total synthesis of vanchrobactin (**43**) (Scheme 8). 2,3-dihydroxybenzoic acid (2,3-DHBA) was first protected by benzyl bromide to generate **1b**. Subsequent saponification with barium hydroxide gave 2,3-dibenzyloxybenzoic acid **2b**. On the other hand, *N^δ^*-Cbz-d-ornithine-OMe **3b** was obtained from the Cbz analogue of d-ornithine copper complex. Coupling of **3b** with 2,3-dibenzyloxybenzoic acid **2b** gave compound **4b**. Deprotection of the benzyl groups in **5b**, introduction of the guanidine functionality to obtain **6b** and acidic hydrolysis produced vanchrobactin (**43**). Moreover, in order to further deduce structure–activity relationships of vanchrobactin (**43**), several **43** analogues were also synthesized by a similar strategy of the total synthesis of vanchrobactin (**43**) [88]. The results suggest the aromatic ring in catecholate siderophores is essential for the binding of the outer membrane receptors. The lack of stereochemistry of the amino acid scaffold will affect the siderophore activity in *V. anguillarum*. Moreover, microbes are not always selective in the use of siderophores, they can utilize several different siderophores to transport Fe(III) into microbial cells, even siderophores with very different structures. The relatively low specificity of siderophore utilization may facilitate the design of drugs against different pathogens.

### 4.3. Petrobactin

**Scheme 9:**
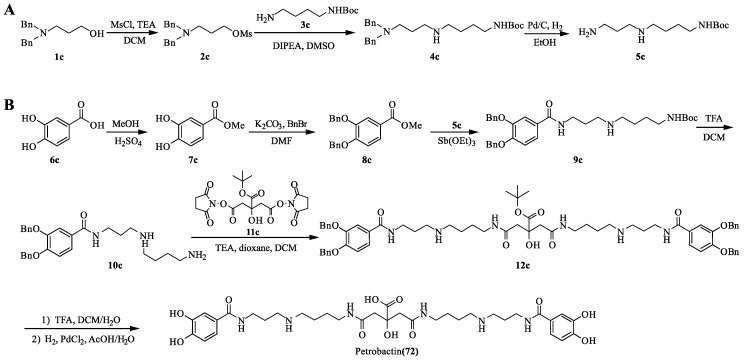
Chemical synthesis of petrobactin (**72**).

Pandey R.K. et al. [89] performed efficient total synthesis of petrobactin **(72)** via antimony triethoxide mediated coupling (Scheme 9). It not only proved the structure of **72**, but also provided sufficient yield for subsequent use in the therapeutic study. First, the hydroxyl group of *N*-dibenzyl-aminoalcohol **1c** was mesyl protected to provide compound **2c**. Nucleophilic displacement of O-Ms with amine **3c** yielded **4c**. Debenzylation of **4c** under H_2_-Pd/C conditions to yield **5c**. On the other hand, esterification of **6c** under reflux conditions was performed, followed by benzyl protection of catechol unit to yield **8c**. Ester–amide exchange was performed with amine **5c** and acid **8c** to produce **9c**. The *N*-Boc group was removed from **9c** to obtain amine **10c**. Coupling of **10c** and **12c** was carried out using trimethylamine to yield protected petrobactin **12c**. Finally, **12c** was debenzylated to yield petrobactin (**72**)**.** Moreover, sidechain-modified petrobactin derivatives were also synthesized for the study of iron binding, the results suggest both acylation and alkylation of the spermidine sidechain basically do not affect iron binding properties of the siderophore [90].

## 5. Conclusions and Perspectives

Siderophores from marine microbes are an important class of secondary metabolites that have intrigued microbiologists, chemists, and biochemists due to their structural diversity and potential applications in diseases and medicinal agents. However, with widely stated estimate that less than 1% marine microbes have been brought culture [91], the great challenge includes the development of new methods to bring more marine microbes into culture. Moreover, further detailed understanding of chemical synthesis and biosynthesis of marine siderophores will provide valuable insight for their study of biological activities, and elucidation of mechanism. In total, marine siderophore research will continue stimulating a multidisciplinary research effort that will facilitate their potential applications in medicine and health care.

## Figures and Tables

**Table 1:** List of abbreviations.

Abbreviation	Full Name
CAS	Chrome azurol sulfonate
ED_50_	Median effective dose
IC_50_	Hablalf maximal inhibition concentration
NRPSs	Nonribosomal peptide synthetases
HSC	*N*-hydroxy-*N*-succinylcadaverine
HSDs	*N*-hydroxy-*N*-succinyl diamines
ArCP	Aryl carrier protein
Cy	Cyclisation
PCP	Peptidyl carrier protein
NIS	NRPS-independent siderophore
hLys	*N^6^*-hydroxy-l-lysine
ahLys	*N^6^*-acetyl-*N^6^*-hydroxy-l-lysine
3-DHS	3-dehydroshikimate
3,4-DHBA	3,4-dihydroxybenzoic acid
2,3-DHBA	2,3-dihydroxybenzoic acid

**Table 3:** The EC numbers of enzymes.

Enzymes	EC Numbers
EnzA	4.1.1.7
VabC	3.1.22.4
VabD	2.7.8.7
VabF	3.2.1.55
Irp1	2.3.2.27 4.2.1.3
Irp2	2.3.2.27
Irp3	2.3.2.27
Irp4	2.3.2.27
AngB	3.3.2.1
AngE	1.14.14.148
VabD	2.7.8.7
IucA	6.3.2.38
IucC	6.3.2.39
IucD	1.14.13.59
AsbF	4.2.1.118

## References

[1] Chen J.W., Wang B.X., Lu Y.J., Guo Y.Q., Sun J.D., Wei B., Zhang H.W., Wang H. (2019). Quorum sensing inhibitors from marine microorganisms and their synthetic derivatives. Mar. Drugs.

[2] Liu X., Ashforth E., Ren B., Song F., Dai H., Liu M., Wang J., Xie Q., Zhang L. (2010). Bioprospecting microbial natural product libraries from the marine environment for drug discovery. J. Antibiot..

[3] Chen J.W., Wu Q.H., Rowley D.C., Al-Kareef A.M., Wang H. (2015). Anticancer agent-based marine natural products and related compounds. J. Asian Nat. Prod. Res..

[4] Gledhill M., Buck K.N. (2012). The organic complexation of iron in the marine environment: A review. Front. Microbiol..

[5] Vraspir J.M., Butler A. (2009). Chemistry of marine ligands and siderophores. Ann. Rev. Mar. Sci..

[6] Butler A. (2005). Marine siderophores and microbial iron mobilization. Biometals.

[7] Paul A., Dubey R. (2014). Characterization of protein involved in nitrogen fixation and estimation of cofactor. Intl. J. Adv. Biotech. Res..

[8] Wencewicz T.A., Long T.E., Mollmann U., Miller M.J. (2013). Trihydroxamate siderophore-fluoroquinolone conjugates are selective sideromycin antibiotics that target *Staphylococcus aureus*. Bioconjugate Chem..

[9] Schalk I.J. (2018). A trojan-horse strategy including a bacterial suicide action for the efficient use of a specific Gram-positive antibiotic on Gram-negative bacteria. J. Med. Chem..

[10] Miethke M., Marahiel M.A. (2007). Siderophore-based iron acquisition and pathogen control. Microbiol. Mol. Biol. Rev..

[11] Page M.G. (2013). Siderophore conjugates. Ann. N. Y. Acad. Sci..

[12] Souto A., Montaos M.A., Balado M., Osorio C.R., Rodríguez J., Lemos M.L., Jiménez C. (2013). Synthesis and antibacterial activity of conjugates between norfloxacin and analogues of the siderophore vanchrobactin. Bioorg. Med. Chem..

[13] Wencewicz T.A., Miller M.J. (2013). Biscatecholate-monohydroxamate mixed ligand siderophore- carbacephalosporin conjugates are selective sideromycin antibiotics that target *Acinetobacter baumannii*. J. Med. Chem..

[14] Hamilton J.L., Hatef A., ul-haq M.I., Nair N., Unniappan S., Kizhakkedathu J.N. (2014). Clinically approved iron chelators influence zebrafish mortality, hatching morphology and cardiac function. PloS ONE.

[15] Guan L.L., Kamino K. (2001). Bacterial response to siderophore and quorum-sensing chemical signals in the seawater microbial community. Bmc Microbiol..

[16] Popat R., Harrison F., Da S.A., Easton S.A., Mcnally L., Williams P., Diggle S.P. (2017). Environmental modification via a quorum sensing molecule influences the social landscape of siderophore production. P. Roy. Soc. B Biol. Sci..

[17] Lamont I.L., Beare P.A., Urs O., Vasil A.I., Vasil M.L. (2002). Siderophore-mediated signaling regulates virulence factor production in *Pseudomonas aeruginosa*. Proc. Natl. Acad. Sci. USA.

[18] Baakza A., Vala A., Dave B., Dube H. (2004). A comparative study of siderophore production by fungi from marine and terrestrial habitats. J. Exp. Mar. Biol. Ecol..

[19] Saha M., Sarkar S., Sarkar B., Sharma B., Bhattacharjee S., Tribedi P. (2016). Microbial siderophores and their potential applications: a review. Environ. Sci. Pollut. Res. Int..

[20] Moriah S., Alison B. (2009). Microbial iron acquisition: marine and terrestrial siderophores. Chem. Rev..

[21] Khan A., Singh P., Srivastava A. (2018). Synthesis, nature and utility of universal iron chelator-siderophore: a review. Microbiol. Res..

[22] Gauglitz J.M., Akira I., Yusai I., Alison B. (2014). Microbial tailoring of acyl peptidic siderophores. Biochemistry.

[23] Martinez J.S., Zhang G.P., Holt P.D., Jung H.T., Carrano C.J., Haygood M.G., Butler A. (2000). Self-assembling amphiphilic siderophores from marine bacteria. Science.

[24] Martinez J.S., Carter-Franklin J.N., Mann E.L., Martin J.D., Haygood M.G., Butler A. (2003). Structure and membrane affinity of a suite of amphiphilic siderophores produced by a marine bacterium. Proc. Natl. Acad. Sci. USA.

[25] Gauglitz J.M., Butler A. (2013). Amino acid variability in the peptide composition of a suite of amphiphilic peptide siderophores from an open ocean *Vibrio* species. J. Biol. Inorg. Chem..

[26] Walker L.R., Tfaily M.M., Shaw J.B., Hess N.J., Paša-Tolić L., Koppenaal D.W. (2017). Unambiguous identification and discovery of bacterial siderophores by direct injection 21 Tesla Fourier transform ion cyclotron resonance mass spectrometry. Metallomics.

[27] Gilis A., Khan M.A., Cornelis P., Meyer J.M., Mergeay M., Lelie D.V.D. (1996). Siderophore-mediated iron uptake in *Alcaligenes eutrophus* CH34 and identification of *aleB* encoding the ferric iron-alcaligin E receptor. J. Bacterial..

[28] Takahashi A., Nakamura H., Kameyama T., Kurasawa S., Naganawa H., Okami Y., Takeuchi T., Umezawa H., Iitaka Y. (1987). Bisucaberin, a new siderophore, sensitizing tumor cells to macrophage-mediated cytolysis. II. Physico-chemical properties and structure determination. J. Antibiot..

[29] Fujita M.J., Sakai R. (2014). Production of avaroferrin and putrebactin by heterologous expression of a deep-sea metagenomic DNA. Mar. Drugs.

[30] Nadia K., Simon A., Song L.J., Daniel O.C., Gregory L.C. (2008). Identification of a gene cluster that directs putrebactin biosynthesis in *Shewanella* species: PubC catalyzes cyclodimerization of N-hydroxy-N-succinylputrescine. J. Am. Chem. Soc..

[31] Moore C.H., Foster L., Gerbig D.G., Dyer D.W., Gibson B.W. (1995). Identification of alcaligin as the siderophore produced by *Bordetella pertussis* and *B. bronchiseptica*. J. Bacteriol..

[32] Kameyama T., Takahashi A., Kurasawa S., Ishizuka M., Okami Y., Takeuchi T., Umezawa H. (1987). Bisucaberin, a new siderophore, sensitizing tumor cells to macrophage-mediated cytolysis. I. Taxonomy of the producing organism, isolation and biological properties. J. Antibiot..

[33] Yoshida T., Hayashi K.I., Ohmoto H. (2002). Dissolution of iron hydroxides by marine bacterial siderophore. Chem. Geol..

[34] Masaki J.F., Koji N., Ryuichi S. (2013). Bisucaberin B, a linear hydroxamate class siderophore from the marine bacterium *Tenacibaculum mesophilum*. Molecules.

[35] Zhang F., Barns K., Hoffmann F.M., Braun D.R., Andes D.R., Bugni T.S. (2017). Thalassosamide, a siderophore discovered from the marine-derived bacterium, *Thalassospira profundimaris*. J. Nat. Prod..

[36] Takehana Y., Umekita M., Hatano M., Kato C., Sawa R., Igarashi M. (2017). Fradiamine A, a new siderophore from the deep-sea actinomycete *Streptomyces fradiae* MM456M-mF7. J. Antibiot..

[37] Wu Q., Deering R.W., Zhang G., Wang B., Li X., Sun J., Chen J., Zhang H., Rowley D.C., Wang H. (2018). Albisporachelin, a new hydroxamate type siderophore from the deep ocean sediment-derived actinomycete *Amycolatopsis albispora* WP1^T^. Mar. Drugs.

[38] Ejje N., Soe C.Z., Gu J., Codd R. (2013). The variable hydroxamic acid siderophore metabolome of the marine actinomycete *Salinispora tropica* CNB-440. Metallomics.

[39] Amin S.A., Green D.H., Küpper F.C., Carrano C.J. (2009). Vibrioferrin, an unusual marine siderophore: iron binding, photochemistry, and biological implications. Inorg. Chem..

[40] Nielsen A., Mansson M., Wietz M., Varming A.N., Phipps R.K., Larsen T.O., Gram L., Ingmer H. (2012). Nigribactin, a novel siderophore from *Vibrio nigripulchritudo*, modulates *Staphylococcus aureus* virulence gene expression. Mar. Drugs.

[41] Soengas R.G., Anta C., Espada A., Paz V., Ares I.R., Balado M., Rodríguez J., Lemos M.L., Jiménez C. (2006). Structural characterization of vanchrobactin, a new catechol siderophore produced by the fish pathogen *Vibrio anguillarum* serotype O2. Tetrahedron Lett..

[42] Balado M., Osorio C.R., Lemos M.L. (2008). Biosynthetic and regulatory elements involved in the production of the siderophore vanchrobactin in *Vibrio anguillarum*. Microbiol..

[43] Balado M., Osorio C.R., Lemos M.L. (2006). A gene cluster involved in the biosynthesis of vanchrobactin, a chromosome-encoded siderophore produced by *Vibrio anguillarum*. Microbiol..

[44] Iglesias E., Brandariz I., Jiménez C., Soengas R.G. (2011). Iron(III) complexation by vanchrobactin, a siderophore of the bacterial fish pathogen *Vibrio anguillarum*. Metallomics.

[45] Moriah S., Andrew H., John B., Murray M., Margo H., Alison B. (2010). Vanchrobactin and anguibactin siderophores produced by *Vibrio* sp. DS40M4. J. Nat. Prod..

[46] Han A.W., Sandy M., Fishman B., Trindade-Silva A.E., Soares C.A.G., Distel D.L., Butler A., Haygood M.G. (2013). Turnerbactin, a novel triscatecholate siderophore from the shipworm endosymbiont *Teredinibacter turnerae* T7901. PLoS ONE.

[47] Matsuo Y., Kanoh K., Jang J.H., Adachi K., Matsuda S., Miki O., Kato T., Shizuri Y. (2011). Streptobactin, a tricatechol-type siderophore from marine-derived *Streptomyces* sp. YM5-799. J. Nat. Prod..

[48] Capon R.J., Stewart M., Ratnayake R., Lacey E., Gill J.H. (2007). Citromycetins and Bilains A-C: new aromatic polyketides and diketopiperazines from Australian marine-derived and terrestrial *Penicillium* spp.. J. Nat. Prod..

[49] Lee I.K., Ki D.W., Kim S.E., Yeom J.H., Kim Y.S., Yun B.S. (2011). Pistillarin salt, a dicatecholspermidine family member from *Gomphus floccosus*, inhibits DNA single strand breakage by the fenton reaction. J. Korean Soc. Appl. Biol..

[50] Hider R.C., Kong X. (2010). Chemistry and biology of siderophores. Nat. Prod. Rep..

[51] Homann V.V., Sandy M., Tincu J.A., Templeton A.S., Tebo B.M., Butler A. (2009). Loihichelins A-F, a suite of amphiphilic siderophores produced by the marine bacterium *Halomonas* LOB-5. J. Nat. Prod..

[52] Vraspir J.M., Holt P.D., Butler A. (2011). Identification of new members within suites of amphiphilic marine siderophores. Biometals.

[53] Andrew W., Nicholas G., Logan W., Hebelin C., Brad H., Douglas H., Russell G. (2018). Isolation of imaqobactin, an amphiphilic siderophore from the Arctic marine bacterium *Variovorax* Species RKJM285. J. Nat. Prod..

[54] Haygood M.G., Holt P.D., Butler A. (1993). Aerobactin production by a planktonic marine *Vibrio* sp.. Limnol. Oceanogr..

[55] Fuse H., Murakami K., Takimura O., Kamimura K., Yamaoka Y. (1998). Phylogenetic analysis of marine environmental strains of *Vibrio* that produce aerobactin. J. Mar. Biotechnol..

[56] Martin J.D., Ito Y., Homann V.V., Haygood M.G., Butler A. (2006). Structure and membrane affinity of new amphiphilic siderophores produced by *Ochrobactrum* sp. SP18. J. Biol. Inorg. Chem..

[57] Boiteau R.M., Repeta D.J. (2015). An extended siderophore suite from *Synechococcus* sp. PCC 7002 revealed by LC-ICPMS-ESIMS. Metallomics.

[58] Barbeau K., Zhang G., Live D.H., Butler A. (2002). Petrobactin, a photoreactive siderophore produced by the oil-degrading marine bacterium *Marinobacter hydrocarbonoclasticus*. J. Am. Chem. Soc..

[59] Hickford S.J., Kupper F.G., Carrano C.J., Blunt J.W., Butler A. (2004). Petrobactin sulfonate, a new siderophore produced by the marine bacterium *Marinobacter hydrocarbonoclasticus*. J. Nat. Prod..

[60] Lee J.Y., Janes B.K., Passalacqua K.D., Pfleger B.F., Bergman N.H., Liu H., Håkansson K., Somu R.V., Aldrich C.C., Cendrowski S. (2007). Biosynthetic analysis of the petrobactin siderophore pathway from *Bacillus anthracis*. J. Bacteriol..

[61] Holt P.D., Reid R.R., Lewis B.L., Luther G.W., Alison B. (2005). Iron(III) coordination chemistry of alterobactin A: a siderophore from the marine bacterium *Alteromonas luteoviolacea*. Inorg. Chem..

[62] Reid R.T., Livet D.H., Faulkner D.J., Butler A. (1993). A siderophore from a marine bacterium with an exceptional ferric ion affinity constant. Nature.

[63] Kaneo K., Kei K., Guan L., Kyoko A., Yoshikazu S. (2004). Pseudoalterobactin A and B, new siderophores excreted by marine bacterium *Pseudoalteromonas* sp. KP20-4. J. Antibiot..

[64] Hannah K., Alison B. (2013). Isolation, structure elucidation, and iron-binding properties of lystabactins, siderophores isolated from a marine *Pseudoalteromonas* sp.. J. Nat. Prod..

[65] Souto A., Montaos M.A., Rivas A.J., Balado M., Osorio C.R., Rodríguez J., Lemos M.L., Jiménez C. (2012). Structure and biosynthetic assembly of piscibactin, a siderophore from *Photobacterium damselae* subsp. *piscicida*, predicted from genome analysis. Eur. J. Org. Chem..

[66] Balado M., Benzekri H., Labella A.M., Claros M.G., Manchado M., Borrego J.J., Osorio C.R., Lemos M.L. (2017). Genomic analysis of the marine fish pathogen *Photobacterium damselae* subsp. *piscicida*: Insertion sequences proliferation is associated with chromosomal reorganisations and rampant gene decay. Infect. Genet. Evol..

[67] Balado M., Lages M.A., Fuentes-Monteverde J.C., Martinez-MatamorosJaime D., Rodriguez J., Jiménez C., Lemos M.L. (2018). The siderophore piscibactin is a relevant virulence factor for *vibrio anguillarum* favored at low temperatures. Front. Microbiol..

[68] Liu N., Shang F., Xi L., Huang Y. (2013). Tetroazolemycins A and B, two new oxazole-thiazole siderophores from deep-sea *Streptomyces olivaceus* FXJ8.012. Mar. Drugs.

[69] Kadi N., Oves-Costales D., Barona-Gomez F., Challis G.L. (2007). A new family of ATP-dependent oligomerization-macrocyclization biocatalysts. Nat. Chem. Biol..

[70] Fujita M.J., Kimura N., Yokose H., Otsuka M. (2012). Heterologous production of bisucaberin using a biosynthetic gene cluster cloned from a deep sea metagenome. Mol. Biosyst..

[71] Fujita M.J., Nobutada K., Atsushi S., Yoichi I., Tomohiro H., Masami O. (2011). Cloning and heterologous expression of the vibrioferrin biosynthetic gene cluster from a marine metagenomic library. Biosci. Biotech. Bioch..

[72] Naka H., Liu M., Actis L.A., Crosa J.H. (2013). Plasmid- and chromosome-encoded siderophore anguibactin systems found in marine *vibrios*: biosynthesis, transport and evolution. Biometals.

[73] Alice A.F., Lopez C.S., Crosa J.H. (2005). Plasmid- and chromosome-encoded redundant and specific functions are involved in biosynthesis of the siderophore anguibactin in *Vibrio anguillarum* 775: a case of chance and necessity?. J. Bacteriol..

[74] Liu Q., Ma Y., Zhou L., Zhang Y. (2005). Gene cloning, expression and functional characterization of a phosphopantetheinyl transferase from *Vibrio anguillarum* serotype O1. Arch. Microbiol..

[75] Lorenzo M.D., Stork M., Crosa J.H. (2011). Genetic and biochemical analyses of chromosome and plasmid gene homologues encoding ICL and ArCP domains in *Vibrio anguillarum* strain 775. Biometals.

[76] Welch T.J., Chai S., Crosa J.H. (2000). The overlapping *angB* and *angG* genes are encoded within the trans-acting factor region of the virulence plasmid in *Vibrio anguillarum*: essential role in siderophore biosynthesis. J. Bacteriol..

[77] Naka H., Lopez C.S., Crosa J.H. (2008). Reactivation of the vanchrobactin siderophore system of *Vibrio anguillarum* by removal of a chromosomal insertion sequence originated in plasmid pJM1 encoding the anguibactin siderophore system. Environ. Microbiol..

[78] Lorenzo M.D., Poppelaars S., Stork M., Nagasawa M., Tolmasky M.E., Crosa J.H. (2004). A nonribosomal peptide synthetase with a novel domain organization is essential for siderophore biosynthesis in *Vibrio anguillarum*. J. Bacteriol..

[79] Tolmasky M.E., Actis L.A., Crosa J.H. (1995). A histidine decarboxylase gene encoded by the *Vibrio anguillarum* plasmid pJM1 is essential for virulence: histamine is a precursor in the biosynthesis of anguibactin. Mol. Microbiol..

[80] Wang J., Chen G., Muckenthaler M., Galy B., Hentze M.W., Pantopoulos K. (2004). Iron-mediated degradation of Irp2, an unexpected pathway involving a 2-oxoglutarate-dependent oxygenase activity. Mol. Cell. Biol..

[81] Macheroux P., Plattner H.J., Romaguera A., Diekmann H. (1993). FAD and substrate analogs as probes for lysine *N*^6^-hydroxylase from *Escherichia coli* EN 222. Eur. J. Biochem..

[82] Coy M., Paw B.H., Bindereif A., Neilands J.B. (1986). Isolation and properties of N epsilon-hydroxylysine: acetyl coenzyme A N epsilon-transacetylase from *Escherichia coli* pABN11. Biochemistry.

[83] Bailey D.C., Alexander E., Rice M.R., Drake E.J., Mydy L.S., Aldrich C.C., Gulick A.M. (2018). Structural and functional delineation of aerobactin biosynthesis in hypervirulent *Klebsiella pneumoniae*. J. Biol. Chem..

[84] Pfleger B.F., Kim Y., Nusca T.D., Maltseva N., Lee J.Y., Scaglione J.B., Janes B.K., Anderson E.C., Bergman N.H., Hanna P.C. (2008). Structural and functional analysis of AsbF: origin of the stealth 3,4-dihydroxybenzoic acid subunit for petrobactin biosynthesis. Proc. Natl. Acad. Sci. USA.

[85] Ovescostales D., Kadi N., Fogg M.J., Song L., Wilson K.S., Challis G.L. (2008). Petrobactin biosynthesis: AsbB catalyzes condensation of spermidine with *N^8^*-citryl-spermidine and its *N^1^*-(3,4-dihydroxybenzoyl) derivative. Chem. Commun..

[86] Bergeron R.J., Mcmanis J.S., Perumal P.T., Algee S.E. (1991). The total synthesis of alcaligin. J. Org. Chem..

[87] Soengas R.G., Anta C., Espada A., Nieto R.M., Larrosa M., Rodríguez J., Jiménez C. (2007). Vanchrobactin: absolute configuration and total synthesis. Tetrahedron Lett..

[88] Soengas R.G., Larrosa M., Balado M., Rodriguez J., Lemos M.L., Jimenez C. (2008). Synthesis and biological activity of analogues of vanchrobactin, a siderophore from *Vibrio anguillarum* serotype O2. Org. Biomol. Chem..

[89] Pandey R.K., Jarvis G.G., Low P.S. (2012). Efficient synthesis of the siderophore petrobactin via antimoy triethoxide mediated coupling. Tetrahedron Lett..

[90] Bugdahn N., Oberthür M. (2014). Syntheses and iron binding affinities of the *Bacillus anthracis* siderophore petrobactin and sidechain-modified analogues. Eur. J. Org. Chem..

[91] Blunt J.W., Copp B.R., Keyzers R.A., Munro M.H., Prinsep M.R. (2016). Marine natural products. Nat. Prod. Rep..

